# Multiomics assessment of lung adenocarcinoma subtypes defined through tumor purity-adjusted DNA methylation

**DOI:** 10.1186/s13073-026-01609-x

**Published:** 2026-02-14

**Authors:** Deborah F. Nacer, Elsa Arbajian, Srinivas Veerla, Mattias Aine, Mats Jönsson, Frida Rosengren, Anna Karlsson, Annette Salomonsson, Sofi Isaksson, Maria Planck, Johan Staaf

**Affiliations:** 1 Division of Translational Cancer Research, Department of Laboratory Medicine, Lund University Cancer Center, Lund University, Medicon Village, Lund, SE-22381 Sweden; 2 Division of Oncology, Department of Clinical Sciences Lund, Lund University Cancer Center, Lund University, Medicon Village, Lund, 22381 Sweden

**Keywords:** Epigenetics, Primary lung cancer, Subclassification, Tumor intrinsic patterns

## Abstract

**Background:**

Molecular subtypes of lung adenocarcinoma (LUAD) with varying prognosis and characteristics have been proposed based on one or two-dimensional studies but are not yet implemented into clinical routine. Epigenetic modifications in cancer cells are independent of sequence variants, directly linked to gene and genome regulation, and thus provide important information to guide subclassification efforts.

**Methods:**

We performed in-depth epigenomic profiling of 95 primary LUAD samples from a Swedish discovery cohort with comprehensive clinicopathological, epigenomic, genomic, transcriptomic, proteomic, and metabolomic data. Additionally, we estimated pure tumor cell methylomes using a computational approach. We subdivided the discovery cohort into four epigenetic subtypes, the epitypes, reflecting distinct tumor cell methylation states. Resulting epitypes were contrasted based on clinicopathological and molecular features, and our main findings were validated in two additional primary tumor cohorts totaling over 700 samples.

**Results:**

Of the four DNA methylation epitypes, M1-M4, M1 and M4 were associated with the previously proposed mRNA subtypes Terminal Respiratory Unit and Proximal Proliferative, respectively. Epitypes M2 and M3 showed similar mRNA/protein subtype composition but differed with respect to e.g., higher expression of the LUAD histology-associated NAPSA/surfactant metabolism expression metagene in M3. Genes included in this metagene showed lower DNA methylation in M3, counter to a global tendency towards promoter hypermethylation in this epitype. To further delineate tumor intrinsic links between the epigenomic and expression phenotypes, 62 LUAD cell lines classified into the four epitypes were investigated and recapitulated several characteristics from the tumor epitypes, such as methylation and expression pattens of NAPSA/surfactant genes, highlighting epigenetic states as likely drivers or maintainers of broad tumor phenotypes and differentiation states.

**Conclusions:**

Dissecting LUAD based on combined biological characteristics using multiomics data has deepened our understanding of the heterogeneity in this complex disease and the mechanisms underlying phenotype formation and maintenance. There remains a critical need for large, publicly accessible, well-annotated multiomic LUAD cohorts to support rigorous subtype discovery and validation, particularly those linked to targeted therapy trial outcomes.

**Supplementary Information:**

The online version contains supplementary material available at 10.1186/s13073-026-01609-x.

## Background

Lung adenocarcinoma (LUAD) is one of the major histologic subtypes of lung cancer and its incidence is increasing, especially among women [1, 2]. There is heterogeneity within this disease, and patients with LUAD differ in prognosis and present a range of clinicopathological and tumor characteristics that include selected treatment predictive alterations. Clinical differentiation between subtypes of LUAD alongside patient stratification based on molecular characteristics has the potential for treatment optimization, improving patient survival and quality of life [3] as seen in e.g., breast cancer [4, 5]. Despite not being presently used in the clinical setting, subtypes of primary LUAD have been proposed based on different clinicopathological and molecular characteristics [6–12], most prominently using gene expression data. Moreover, recurring patterns have been observed across the different subdivisions such as the association between no smoking history, less aggressive tumors, specific transcriptional programs, and variants in the *EGFR* gene [3, 6, 7, 9, 13].

Irrespective of the genomic profiling method (-omics) used as base for studies, molecular subdivision of LUAD and other cancer types has typically been performed using bulk tissue samples, i.e., samples including both malignant and non-malignant cells (such as immune cells) that interact with the tumor either facilitating or hindering its progression. The presence of non-tumor cells can act as a confounder in subtyping studies, irrespective of omics method, as the generated output will inherently represent a mixture of intrinsic tumor characteristics together with characteristics of non-malignant cells in the tumor microenvironment (TME). The extent of non-malignant cell influence is guided by the proportion of tumor compared to non-tumor cells in the sample tissue and the exact cell type composition. Methods have been proposed to deal with this issue by e.g., estimating sample purity [14, 15] or deconvolving data to identify which cell types are present in the sample [16–19]. An alternative approach relies on correcting measurements to reflect signals originating only from tumor cells, and such methods have been introduced for copy number data [20] and for genome-wide epigenetic profiling data like DNA methylation arrays [21, 22].

Epigenetic alterations impact gene and genome regulation and are an enabling characteristic of tumor formation [23]. Epigenetic patterns, more specifically DNA methylation patterns, are slowly being elucidated in LUAD through analyses of gene-specific and genome-wide data using bulk tissue samples (e.g., [8, 24]). However, molecular subtyping efforts using epigenetic data purified of normal cell influence could allow for new information on LUAD subtypes to emerge. Here we use DNA methylation data adjusted to reflect values of tumor cells alone to divide 95 primary LUAD samples into four clusters while also taking into consideration that DNA methylation patterns depend on the genetic context being analyzed (e.g., whether CpGs are in gene promoters or not [25]). We contrast and characterize resulting clusters (or epigenetic subtypes, epitypes) using different omics data obtained from the same sets of samples, demonstrating similarities with other subtyping approaches based on gene expression and protein data, as well as present new findings identified with this novel approach. We proceed to validate patterns in two larger cohorts totaling more than 700 primary tumor samples and explore an additional 62 cell lines to distinguish characteristics that seem to be tumor intrinsic. Taken together, there is still considerable tumor heterogeneity left to dissect with respect to molecular subtypes in LUAD when accounting for tumor tissue heterogeneity in bulk tumor analyses. Here, epigenetics may represent a valuable approach to gain new insights into the molecular landscape and our understanding of LUAD as an entity.

## Methods

### Discovery cohort

A total of 95 LUAD samples resected from patients that underwent surgery at the Skåne University Hospital in Lund, Sweden composed the discovery cohort, also referred to as the SUH cohort. Patients had mainly early-stage, primary tumors in the lung, but one patient was classified as having stage IV disease after surgery had been performed (Table 1, Additional file 1: Table S1). The study was approved by the Regional Ethical Review Board in Lund, Sweden (registration number 2004/762). Clinicopathological information was retrieved from Karlsson et al. [26]. Treatment information was only available as whether patients had received adjuvant therapy, and most patients had not (62/73 patients with information, 85%). Data from several genomic profiling methods (-omics) were available for most patients (Table 1) and retrieved/generated as described below.

**Table 1:** Cohorts’ characteristics and omics availability. Clinicopathological characteristics of patients in the included cohorts and omics availability. Displayed are number of samples (except for age at diagnosis) with percentage of total samples between parentheses

		Cohort		
		SUH (*n *= 95)	TCGA (*n* = 418)	Sandoval (*n* = 322)
Use		Discovery	Validation	Validation
Patient sex				
Male		41 (43.2%)	197 (47.1%)	161 (50.0%)
Female		54 (56.8%)	221 (52.9%)	161 (50.0%)
Patient age at diagnosis (years)				
Median		67.0	66.0	65.0
Min—max		36.0—82.8	39.0—88.0	40.0—90.0
Patient smoking status				
Smoker, current		51 (53.7%)	99 (23.7%)	254(78.9%)*
Smoker, former		23 (24.2%)	248 (59.3%)	
Never smoker		18 (18.9%)	59 (14.1%)	43 (13.4%)
Missing		3 (3.2%)	12 (2.9%)	25 (7.7%)
Tumor stage				
I		66 (69.5%)	230 (55.0%)	181 (56.2%)
II		16 (16.8%)	101 (24.2%)	53 (16.5%)
III		10 (10.5%)	65 (15.6%)	77 (23.9%)
IV		1 (1.1%)	20 (4.8%)	11 (3.4%)
Missing		2 (2.1%)	2 (0.4%)	-
Data availability				
Epigenomics		95 (100%)	418 (100%)	322 (100%)
Genomics		95 (100%)**	418 (100%)	-
Transcriptomics		95 (100%)	418 (100%)	-
Proteomics		86 (90.5%)	-	-
Metabolomics		37 (38.9%)	-	-

* Both current and former. ** 100% using the 26-gene panel; 85 (89.5%) using the larger 370-gene panel

#### Epigenomics

DNA methylation beta values (representing the methylated/methylated + unmethylated signal ratio for specific CpG sites) generated with the Illumina Infinium HumanMethylation450 BeadChip array were obtained from Karlsson et al. [8] for 68 samples (Gene Expression Omnibus, GEO, GSE60645 [27]) and from Arbajian et al. [28] for 14 samples (GEO, GSE149521 [29]). For the remaining 13 samples without publicly available data, research biopsies were collected after routine pathological evaluation of tumors and stored at –80 °C. The AllPrep DNA/RNA mini kit (Qiagen) was used for DNA extraction following manufacturer’s instructions. Bisulfite conversion was performed using the EZ DNA Methylation kit (Zymo Research) with 250 ng of DNA per sample. The bisulfite converted DNA was eluted in 15 µl following the manufacturer's protocol, evaporated to a volume of < 4 µl, and used for the methylation analysis. DNA methylation profiling was performed with the Illumina Infinium MethylationEPIC v1.0 array by the SNP&SEQ Technology Platform in Uppsala, Sweden (www.genotyping.se). Data were pre-processed as described by Staaf and Aine [21] and have been deposited to GEO (GSE311943 [30]). Additionally, only CpG sites profiled in all samples were kept, and CpGs located in sex chromosomes were excluded from analyses, resulting in 381,355 interrogated CpGs in this cohort.

##### Beta adjustment based on sample tumor purity

As DNA methylation data are generated from signals originating from several different cells present in varying proportions in bulk tissue samples, beta values were adjusted with the statistical method described by Staaf and Aine [21] to reflect values originating from tumor cells only. Briefly, based on the observed linear relationship between DNA methylation beta values and tumor purity estimates, cohort samples are divided into one, two, or three different populations for each CpG in the data set and statistically modelled by regressions. Beta values are then adjusted per sample and per CpG based on the calculated regressions to reflect values of samples composed only of malignant cells (or only of non-malignant cells, data that were not used in our analyses). Purity estimates from genomic data were obtained from Lehtiö et al. [9] for most samples. For samples profiled with the MethylationEPIC array, estimates were calculated using the Tumor Aberration Prediction Suite [31] based on Affymetrix CytoScan HD Arrays and provided together with DNA methylation data. It is important to note that tumor purity estimates obtained from different algorithms tend to be highly correlated [22], and that minor variations in these estimates have little effect on the resulting adjusted beta values [21]. All subsequent analyses were performed on tumor purity-adjusted beta values focusing on malignant cells only.

##### CpG annotation and division into contexts

Custom annotations for included CpGs were created by overlapping the genomic coordinates of CpGs and other information such as regions of open chromatin identified with the Assay for Transposase-Accessible Chromatin using sequencing (ATAC-seq) technique reported by The Cancer Genome Atlas (TCGA) consortium in the TCGA LUAD cohort [32] and transcription factor binding sites (TFBS) for 340 transcription factors (TFs) identified in 129 cell lines collected from ENCODE [33] as described by Staaf and Aine [21]. Relative to genes, CpGs were generally classified as: (i) in gene promoters (*n* = 98,960 CpGs, 25.9%), if they were situated within a 500 base pair (bp) window upstream or downstream from the transcription start site (TSS) of any gene; (ii) proximal to gene promoters (*n* = 91,789, 24.1%), if situated within 5000 bp up- or downstream from any TSSs but not in the promoter window; or (iii) distal (*n* = 190,606, 50.0%), if situated more than 5000 bp away from any TSS. Given that DNA methylation patterns and expected alterations in tumor cells depend on local CpG density and genomic regions [25], we used the annotations to divide CpGs into different methylation contexts based on combinations of chromatin status (open chromatin is also referred to as ATAC peak in this work) and gene coordinates.

##### Sample clustering

**Fig. 1:**
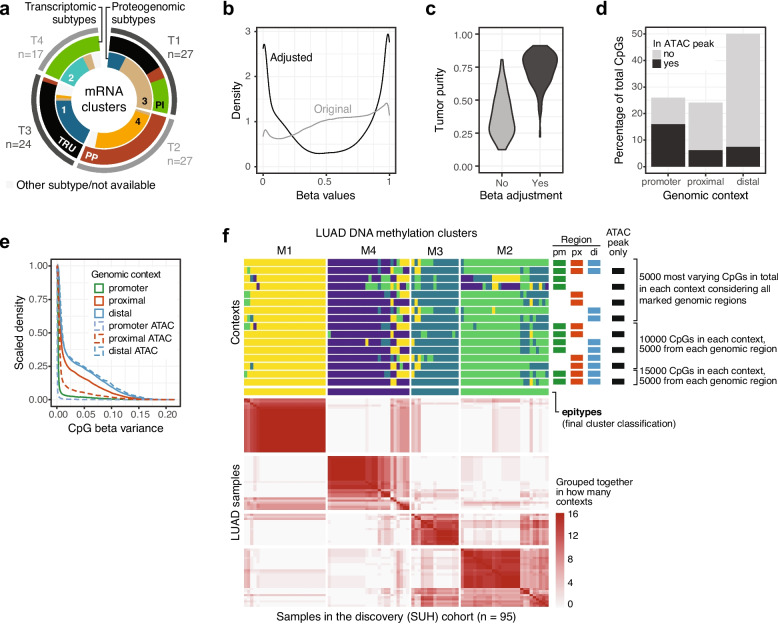
Clustering of samples in the SUH discovery cohort and CpG characteristics. **a** Clusters T1-T4 of size *n* samples defined based on RNA-seq data using the unsupervised SRIQ method greatly overlap with previously proposed gene expression and proteogenomic subtypes. **b** Density of DNA methylation beta values for the 5000 CpGs with highest beta variance in the cohort before (Original) and after adjustment to better reflect values of malignant cells based on tumor purity of samples. **c** Tumor purity for all SUH samples estimated with a cell type deconvolution method based on DNA methylation beta values (MethylCIBERSORT), here performed on unadjusted (as intended) and adjusted beta values. **d** Proportion of CpGs in different genomic regions (promoter, proximal, distal) and how many of them overlap with previously reported regions of open chromatin (i.e., ATAC peaks). **e** Beta variance per context considering genomic regions and chromatin accessibility (ATAC = in ATAC peak, i.e., open chromatin). **f** Sample cluster assignment based on 16 combinations of methylation contexts (above) and how often any two samples were assigned to the same cluster considering all contexts (below) define the final four methylation epitypes (M1-M4). Contexts were defined based on CpG overlap with different gene regions (pm = gene promoter, px = proximal to promoter, di = distal from promoters) and with regions of open chromatin (ATAC peaks) observed in the TCGA breast cancer cohort

To establish the number of LUAD DNA methylation clusters to characterize with multiomics data, we used adjusted beta values of the 5000 CpGs with the highest variance in the data considering all available CpGs and non-negative matrix factorization (NMF) with the NMF [34] R package v0.24.0 (factorization rank ranging from two to eight, 100 runs each). To account for biological information encoded in areas of the genome not represented by the 5000 most varying CpGs in the cohort, we proceeded to independently cluster samples using an additional 15 CpG subsets based on combinations of the DNA methylation contexts described above and the most varying CpGs per context (Fig. 1). A total of 26,592 unique CpGs were used as CpGs could be present in more than one methylation context. NMF classifications were performed as above, but with a fixed factorization rank of four. Sample classifications from the 16 methylation context combinations were considered when deriving the final cluster solution that corresponded to the proposed epigenetic subtypes, or epitypes.

##### Differential methylation analysis

To identify differentially methylated CpGs (DMCs) among all CpGs in the data set, we first performed Kruskal–Wallis tests per CpG to reduce the number of CpGs to analyze to only those that showed differences in beta values for at least one of the epitypes. For CpGs with *p* < 0.05 after multiple testing correction, pairwise comparisons of beta values between epitypes were performed using the Wilcoxon rank sum test, with results again adjusted for multiple testing. To define DMCs in an epitype compared to the other three, we filtered the pairwise comparison results to keep only CpGs that were statistically different between that one epitype and all other three epitypes (adjusted *p*-value < 0.05), i.e., three significant pairwise comparisons involving the same epitype. Additionally, the beta median in the epitype associated with a DMC had to be consistently higher or lower than the medians of the other epitypes, and a minimum difference in median beta value of 0.3 between the epitypes was also required.

##### Linking DMCs to genes

To connect CpGs to specific genes, we calculated the Pearson correlation between beta values and gene expression (log2 of fragments per kilobase million, FPKM) of DMC-gene pairs identified based on genomic windows of 800,000 bp centered around TSSs of genes using the findOverlaps() function of the GenomicRanges [35] R package v1.48.0. The choice of 400,000 bp up- and downstream of each gene was motivated by this distance being slightly greater than the average gene-enhancer separation calculated from 5349 unique enhancers and their target genes in lung tissue reported in the Human enhancer RNA Atlas [36]. TSS coordinates were retrieved from GENCODE hg38 rel21 annotation files and filtered to only keep those that had gene expression available with a 95^th^ percentile above zero to exclude low varying genes in the data set. Only negative correlations with *p*-values < 0.05 after Benjamini-Hochberg (BH) adjustment were kept. DMC and genes showed a many-to-many relationship, i.e., a DMC could be inside the window of many genes and a gene could have many DMCs in its window. Genes were associated to epitypes based on to which epitype the DMC in the DMC-gene pair was associated. Gene set overrepresentation analyses (GSOA) using the resulting genes were performed using the enricher() function from the clusterProfiler [37] R package v4.4.4 and combined gene sets from Reactome (1615 sets), KEGG (186 sets), and Gene Ontology – Biological Processes (7658 sets) obtained from the msigdbr R package v7.5.1 (https://igordot.github.io/msigdbr/) filtered to contain only genes initially available in unfiltered data.

##### DNA methylation status of specific genes

DNA methylation of specific genes was assessed through analyses of CpGs located within genomic windows centered on genes’ TSSs. Windows were either 12,000 bp wide (centered on the TSS) or 2500 bp wide (2000 bp upstream and 500 bp downstream from the TSS) as specified in different analyses. TSS coordinates were retrieved with the biomaRt [38] R package v2.52.0 with the useMart(biomart = "ensembl", dataset = "hsapiens_gene_ensembl") and getBM() functions to extract attributes or generated as described in [39]. Overlapping CpGs were identified using the GenomicRanges package.

#### Genomics

DNA sequencing data from a custom panel including 370 genes used to detect clinically relevant variants (only putative functional variants retained for analyses) and to estimate tumor mutational burden (TMB) were downloaded from Lehtiö et al. [9] for 85 samples. Additional variant information for 26 genes of interest were downloaded from Karlsson et al. [26] for 84 samples and generated for the remaining 11 samples as described by Karlsson et al. [40] using the Illumina TruSight Tumor panel (Additional file 1: Table S1). Both data sets were combined for the 21 overlapping genes: samples were considered as having a gene variant if one had been detected with any panel.

#### Transcriptomics

The AllPrep DNA/RNA mini kit (Qiagen) was used for extracting 500 ng of RNA following manufacturer’s instructions using fresh frozen samples stored at –80 °C. Libraries were prepared using the Illumina TruSeq stranded mRNA protocol and sequenced on the NovaSeq 6000 system at the Center for Translational Genomics (www.ctg.lu.se) in Lund, Sweden. Demultiplexing was performed using the bcl2fastq2 software v2.18.0.12 (Illumina) with default settings and the quality was checked with FastQC [41] v0.11.3. Reads were mapped to the GRCh38 reference genome using the HISAT2 software [42] v2.1.0 and annotation files from release 103. Finally, RNA-sequencing (RNA-seq) expression data in FPKM were calculated with StringTie [43, 44] v1.3.4d and have been deposited to ArrayExpress (accession number E-MTAB-16082 [45]).

##### Sample clustering

Clustering of discovery samples based on gene expression values was performed using SRIQ [46] on all available transcripts. SRIQ is an unsupervised clustering method that aggregates different concepts such as random forest, quality threshold clustering, and k-nearest neighbors to identify groups of samples or genes without a user-defined optimal number of clusters, and it is available through GitHub (https://github.com/StaafLab/SRIQ). The following parameters were used: 10,000 permutations, 10 iterations, minimum bag size of 1200, and the Pearson method.

##### Calculating expression metagene scores

Rank scores for gene expression-based metagenes (networks of co-expressed genes representing specific biological processes) were calculated per sample as described by Nacer et al. [47]. Briefly, gene transcripts were organized from lower to higher FPKM expression and ranks from all available genes belonging to a metagene were extracted and summed. This way, lower rank scores meant generally lower expression of a metagene when compared to samples with higher rank scores in the same cohort. Six metagenes previously identified through a gene network analysis in lung cancer were used: basal/squamous, napsin A/surfactant (or NAPSA/surfactant), neurodevelopment, immune response, stroma/extra-cellular matrix, and proliferation [8].

##### Differential expression analysis

To identify differentially expressed genes (DEGs) in the epitypes, we removed uninformative gene transcripts by keeping only those with a 95^th^ percentile above 0, i.e., transcripts that had more than 90 samples with an FPKM of 0 were excluded. Remaining transcripts were offset by 0.1, log2-transformed, and median-centered. We then followed the same reasoning and statistical tests as for DMCs to find genes differentially expressed in one epitype when compared to all others. Unlike the DMC analysis, however, no minimum difference between expression means or medians was required. GSOA was performed as described for the DMC analysis with genes separated into eight groups: to which cluster they were associated and whether they were up- or downregulated compared to the other epitypes.

##### Candidate genes for epigenetically regulated expression

Available genes were strictly filtered to keep only those with wider expression (untransformed FPKM range > 100) and DNA methylation (CpG beta range > 0.5) ranges in the data set. Pearson correlation estimates were calculated between remaining genes and CpGs within 6 kbp of their TSSs. Only estimates < −0.25 with p-values adjusted for multiple testing < 0.01 were kept. A gene had to have at least 5 CpGs fulfilling these criteria to be considered a candidate gene in this analysis.

#### Proteomics

Mass spectrometry-based data for almost 10,000 proteins were obtained pre-processed from Lehtiö et al. [9] for 86 samples. Data generation involved tandem mass tag labeling, high-resolution isoelectric focusing prefractionation, and liquid chromatography-mass spectrometry (LC–MS) with data-dependent acquisition as described in the original publication. Differential analysis was performed as described for DEGs.

#### Metabolomics

LC–MS-based data normalized for total ion count for 139 metabolites were obtained from Staaf et al. [48] for 37 samples unevenly distributed between the final epitypes (Additional file 1: Table S1). Differential analysis was performed as described for DEGs after offsetting the data by 10^–9^ and log2-transforming values.

### TCGA validation cohort

Clinicopathological, survival, and data from multiple omics layers were downloaded through the TCGA’s Genomics Data Commons portal following the workflow described by Staaf and Aine [21] for 418 LUAD samples (Table 1, Additional file 1: Table S1), a data set here referred to as the TCGA cohort. Limited treatment information was also obtained – the vast majority of patients had no history of neoadjuvant treatment (*n* = 415, 99%), and most did not receive adjuvant radiotherapy (*n* = 341/392 patients with information, 87%). Any analyses with results reported in this work but not explicitly mentioned below were performed as described in detail for the SUH discovery cohort. Pre-processed HumanMethylation450 DNA methylation data interrogating 421,368 CpG sites were available and adjusted [21] based on tumor purity estimates calculated from whole exome sequencing obtained from Hoadley et al. [49]. Samples were clustered with the same NMF clustering of different methylation context combinations approach as in the discovery cohort but using the most varying CpGs per context considering only TCGA beta values. When comparing gene variant occurrence, only variants of high and moderate impact were considered. A simplified TMB was calculated by summing identified variants (deletions, insertions, single nucleotide polymorphisms) in any genes irrespective of predicted effect as even silent variants are indicative of mutational processes occurring in the genome. Mutational signature exposure for all samples, as made available in the mSignatureDB [50] data base, was retrieved from [51]. RNA-seq data were used to classify samples into the previously proposed Terminal Respiratory Unit (TRU), Proximal Proliferative (PP), or Proximal Inflammatory (PI) gene expression (mRNA) subtypes [6, 7] through nearest centroid classification. RNA-seq data were offset by 0.01, log2-transformed, median-centered, and correlated to reported centroids [6] for each subtype with the Pearson method. The subtype that had the highest correlation value was assigned to a sample. If a sample did not have correlations above the threshold of 0.2, it was considered unclassified.

### Sandoval validation cohort

DNA methylation data (HumanMethylation450 array) from 322 LUAD samples made available by Sandoval et al. [52] were obtained from GEO (GSE39279 [53]) together with patient information (Table 1, Additional file 1: Table S1). No patient had received neoadjuvant treatment, and most patients did not receive any adjuvant therapy (165/219 patients with treatment information, 75%). This data set is here referred to as the Sandoval cohort. As for the TCGA validation cohort, any analyses with results reported in this work but not explicitly mentioned below were performed as described for the SUH discovery cohort. Tumor purities were estimated for Sandoval samples directly from DNA methylation data using the purity_estimation() function of the PureBeta [22] R package. They were then used for beta value adjustment following the rationale from Staaf and Aine [21] as implemented by PureBeta with the reference_based_beta_correction() function and available TCGA LUAD reference regressions. Keeping only CpGs with annotations as described above, adjusted beta values of 411,403 CpGs were available for subsequent analyses. Sample clustering was performed as described for the cohorts above but using the most varying CpGs in the Sandoval cohort alone. Scores for several cell types were estimated through deconvolution of unadjusted DNA methylation data using the lung_NSCLC_adenocarcinoma_v2 signature obtained from the MethylCIBERSORT R package v0.2.0 [18] and the online version of CIBERSORTx [54] (parameters: batch correction and quantile normalization disabled, 1000 permutations, absolute mode). Scores for eight available immune cells (monocytes, eosinophils, neutrophils, B-cells, natural killer cells, cytotoxic T-cells, regulatory T-cells, and helper/effector lymphocytes) were summed to create an immune cell score.

### Publicly available cell line DNA methylation data

Raw DNA methylation (HumanMethylation450 array) idat files from 62 LUAD cell lines (Additional file 1: Table S2) described in Iorio et al. [55] were retrieved from GEO (GSE68379 [56]) and pre-processed as described in Staaf and Aine [21]. No adjustment based on tumor purity estimates was performed as DNA methylation beta values were derived only from tumor cells. Pre-processed RMA normalized expression data of 17,419 genes were also downloaded from online supplementary material of the original publication [55]. Cell lines were classified into the DNA methylation epitypes through Spearman correlation to median beta values of the SUH discovery epitypes. Correlations were calculated separately based on the 5000 most varying CpGs per each of the eight methylation contexts (all/promoter/proximal/distal + open/closed chromatin). Cell lines were assigned to the epitype with highest correlation per context, and the epitype that appears the most across contexts was the final epitype classification of a given cell line, i.e., a majority rule. When there was a tie (four classifications for each epitype), the epitype with highest correlation on average across the four contexts was the assigned one. Alternatively, cell lines were one at a time clustered with the discovery samples based on beta values of the 5000 most varying CpGs in the discovery cohort. This was performed with the NMF R package v0.24.0, a factorization rank (k) of 4, 100 runs, and the same seed set for all cell lines.

### Publicly available single cell RNA-seq (scRNA-seq) data

scRNA-seq data from 82,991 cells representing eight cell types present in the TME of LUAD samples [57], part of the Human Tumor Atlas Network, were downloaded from CZ CELLxGENE [58]. An additional smaller scRNA-seq data set of 4000 cells containing equal proportions of live cells from three LUAD cell lines (NCI-H2228, NCI-H1975, HCC827) was downloaded from GEO (GSE111108 [59, 60]).

### Statistical and survival analyses

All R packages above, as well as any statistical tests mentioned, were executed in an RStudio environment running R v4.2.0. All statistical tests performed were two-sided and p-values were compared to a level of significance of 0.05 unless otherwise specified. The BH method was used for multiple testing correction whenever performed unless otherwise specified. Survival analyses were performed using Kaplan–Meier plots and the log-rank test from the survival v3.4.0 and survminer v0.4.9 R packages. Uni- and multivariate Cox regression was performed with the coxph() function. Clinical endpoints available were overall survival (OS) for the SUH cohort; OS, disease-free interval (DFI), and progression-free interval (PFI) for the TCGA cohort, censored at 10 years follow up; and relapse-free survival (RFS) for the Sandoval cohort.

## Results

### Unsupervised sample clustering using purity-adjusted DNA methylation beta values

The SUH discovery cohort comprised primary LUAD samples from 95 patients (Table 1, Additional file 1: Table S1). We first clustered these samples based on gene expression patterns using a novel unsupervised clustering method (SRIQ [46]) that does not require a pre-selected, reduced number of genes as input nor the specification of a final number of clusters. This returned an optimal number of four clusters in the expression data set (T1-T4) that strongly agree with previously suggested transcriptional [6, 7] and proteomic [9] subtypes of LUAD (Fig. 1a). This indicates that transcriptional and proteomic LUAD subtypes appear well aligned and may, for subtyping purposes, even be considered as partly interchangeable.

As gene regulation can be largely governed by epigenetic patterns, we next turned to global DNA methylation profiling to investigate inter tumor heterogeneity in LUAD. To target epigenomic regulation of only tumor cells, we used a computational method [21] that, based on estimated tumor purity, adjusts bulk DNA methylation data per CpG to account for the influence of non-malignant cells. This adjustment shifted the overall beta distribution (considering all CpGs and all samples) closer to a Bernoulli distribution that better reflects expected biological states of DNA methylation (methylated or not) of a specific cell type (Fig. 1b, Additional file 2: Fig. S1a), and correspondingly increased the estimated tumor fraction of samples when performing cell type deconvolution, consistent with reduced influence from non-malignant cells (Fig. 1c). Importantly, there were no discernible batch effects connected to the different DNA methylation sources forming the discovery cohort in a principal component analysis (Additional file 2: Fig. S1b).

Subtyping efforts commonly focus on the most varying genes and/or CpGs in a data set. Sample classification using NMF clustering of the 5000 most varying CpGs in the data set and varying target number of clusters resulted in four LUAD DNA methylation clusters as the optimal solution for the discovery cohort, and clusters were considerably stable in sample composition across different grouping solutions (Additional file 2: Fig. S1c-d). Varying the number of CpGs did not change sample classification (Additional file 2: Fig. S1e). However, CpGs can be classified into different contexts (depending on e.g., proximity to genes, local CpG densities, chromatin conformation) that appear in different proportions in a data set and show distinct properties such as varying beta variance profiles (Fig. 1d-e). Furthermore, CpGs in different contexts have different baseline methylation status and behave differently in the context of cancer [25]. Therefore, we clustered samples based on adjusted beta values of CpGs divided into eight different methylation contexts and their combinations to allow for more biological traits of tumor cells to be included (Fig. 1f). Based on that only a few samples switched cluster assignment depending on CpG context used, a final clustering solution was derived by calculating how often any two samples were assigned to the same cluster out of all context classifications. These final four methylation clusters (M1-M4) are the epigenetic subtypes, or epitypes, used in subsequent analyses.

### General characteristics of DNA methylation epitypes

**Fig. 2:**
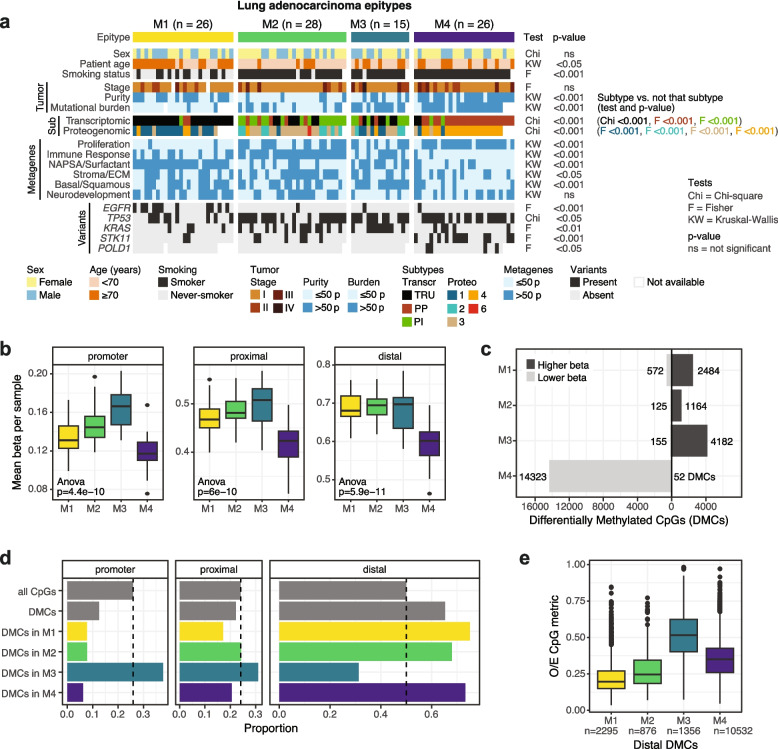
Characteristics of the LUAD DNA methylation epitypes in the discovery cohort. **a** Clinicopathological and molecular characteristics per sample (columns) and p-values of tests performed for statistical significance assessment. Numeric variables like tumor purity, mutational burden, and metagenes are displayed binarized based at the 50^th^ percentile (50 p) cutoff for each variable. **b** Mean beta per sample using all CpGs belonging to a genomic context. **c** Number of differentially methylated CpGs (DMCs) identified across methylation epitypes and whether they show lower or higher beta values compared to the other epitypes. **d** Proportion of CpGs per genomic context considering all available CpGs, all DMCs, and DMCs per M1-M4 epitype. Dashed line = expected proportions based on all CpGs available. **e** Observed over expected (O/E) CpG metric for distal DMCs per epitype indicating the association between M3 DMCs and gene promoters – regions commonly associated with higher CpG density

Defining characteristics of epitypes and statistical significance are shown in Fig. 2a. M1 patients were older when diagnosed with lung cancer and smoked less often, with 58% of them being considered never-smokers (compared to < 8% in other epitypes). Gene expression-based signatures referred to as metagenes were calculated for six biological processes shown to be important in lung cancer [8], revealing highest NAPSA/surfactant scores and lowest cell proliferation in M1. The latter was supported by GSOAs of differential genes and proteins, as well as by lower amino acid availability in this epitype seen with metabolomics data (Additional file 1: Table S3). Additionally, M1 tumors showed a higher proportion of somatic *EGFR* variants (42% vs. 4–7% in M2-M4) and lower proportion of *TP53* variants (35% vs. 68–73% in M2-M4), and they showed the lowest TMB values of all epitypes. Consistent with these findings, M1 was enriched for the previously proposed TRU gene expression subtype and proteogenomic subtype 1.

Unlike the transcriptomic subclassification of the cohort, where all clusters had a clear association with mRNA or protein subtypes of LUAD, DNA methylation epitypes M2 and M3 included several subtypes and harbored most samples with the PI gene expression subtype and proteogenomic subtypes 2 and 3 in the cohort. Despite showing a similar subtype composition to each other, these epitypes differed in several instances. M2 samples had the highest values of the immune response metagene and lower tumor purity, which agrees with more immune cell infiltration in this epitype. This observation was supported by a GSOA of differential genes associated with this epitype through both transcriptomics and proteomics data that returned several terms associated with immune response as significant, such as B-cell activation and T-cell differentiation (Additional file 1: Table S3). In turn, M3 samples showed higher TMB and higher NAPSA/surfactant metagene scores compared to M2. Finally, both M2 and M3 showed higher proportions of tumors with *KRAS* variants (46% and 60% respectively vs. M1 15%, M4 19%), a trait previously associated with the PP subtype and unexpected for these epitypes. Additionally, M3 patients showed worse overall survival when compared to patients from all other epitypes combined (log-rank test, *p* = 0.022).

The M4 epitype was enriched for the PP gene expression subtype and proteogenomic subtype 4. In agreement with what has been shown for these subtypes [6, 7, 9], patients in this epitype did not stand out in any of the analyzed characteristics, but their tumors did: M4 tumor samples showed higher overall TMB (similar to M3), the lowest values of the immune response metagene and a trend of lower expression of genes in the stroma/extracellular matrix signature, as well as lower values of the basal/squamous module, typically associated with lung squamous cell carcinoma. Lower immune response was supported by a GSOA of differential genes less expressed in M4 compared to the other epitypes (Additional file 1: Table S3). Lastly, M4 tumor samples showed higher proportion of somatic variants in the tumor suppressor *STK11* (46% vs. M1 8%, M2 21%, M3 0%), and they were the only ones to show variants in *POLD1* (17% as observed with the extended gene panel data set).

### DNA methylation landscape of LUAD epitypes

Two epitypes exhibited notable generalized DNA methylation patterns: M3 showed promoter hypermethylation and M4 showed global hypomethylation when compared to other epitypes (Fig. 2b). A differential analysis identified 23,023 unique DMCs in the data set that had statistically significant higher or lower beta values in one epitype when compared to the other three (Fig. 2c, Additional file 1: Table S4). Of these, 5316 CpGs (23%) were among the most varying CpGs used for sample clustering. Most DMCs (62%) were linked to M4 and hypomethylated, consistent with the pattern seen across all genomic regions. In general, DMC distribution across genomic regions per epitype was also different than the expected proportion based on all CpGs analyzed (Chi-square test, *p* < 0.001): epitypes showed more DMCs in distal regions and less in gene promoters (Fig. 2d), in line with higher beta variance in the former context (as shown in Fig. 1e).

However, M3 showed the reverse pattern with more DMCs in gene promoters, consistent with the trend of promoter hypermethylation observed in this epitype (Fig. 2b,d). Interestingly, M3 DMCs located in distal elements showed higher values of the observed/expected CpG metric (Fig. 2e), and 55% of them overlapped with CpG islands, two measures of higher CpG density typically associated with gene promoters. To investigate other regulatory elements in the genome, specifically whether any TFs could be affected through differential methylation in a given epitype, we overlapped DMC and TFBS coordinates for 340 TFs collected from ENCODE cell line data. We then compared proportions of CpGs/DMCs per genomic context with and without TFBS overlap per TF. This identified enrichment of four TFs in promoter and proximal DMCs of M3 only (Chi-square tests, *p* < = 0.001 for all): two members of the Polycomb-group (*EZH2* and *SUZ12*), a binding protein (*CREBBP*), and a member of the SMAD family (*SMAD2*).

To explore pathways possibly being differentially epigenetically regulated across M1-M4 epitypes, we performed a correlation analysis between DMC beta values and expression of closely located genes (< 800 kbp genomic window). This resulted in 963 genes of interest for M1, 1225 for M2, 464 for M3, and 3806 for M4 (Additional file 1: Table S4). A GSOA analysis of genes subdivided by whether the DMC displayed higher or lower beta values in the epitype reiterated trends seen before based on e.g., the metagene analysis (Additional file 1: Table S4). For M1, DMCs showed higher beta values in the vicinity of genes connected to several cell cycle terms such as DNA replication, synthesis of DNA, and pyrimidine metabolism. M2 DMCs showed lower DNA methylation values close to genes connected to immune infiltration (positive regulation of leukocyte cell–cell adhesion, antigen processing-cross presentation, positive regulation of immune effector process). The nine terms connected to M3 DMCs related to developmental processes. Finally, M4 DMCs were connected to genes overrepresented in several terms, such as pertaining to the mitochondria (mitochondrial gene expression, translation, and transport), apoptosis (intrinsic apoptotic signaling pathway, apoptotic mitochondrial changes), or metabolism (metabolism of amino acids and derivatives, fatty acid metabolic process), among others.

### DNA methylation of specific pathways and genes differs across epitypes

**Fig. 3:**
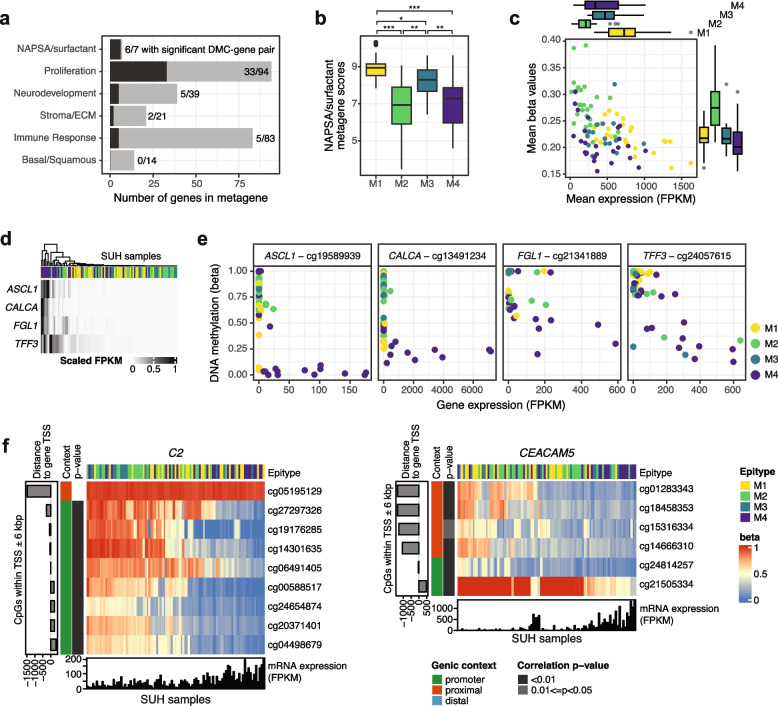
DNA methylation of specific pathways/genes in the discovery cohort. **a** Number of genes included in each mRNA expression metagene and how many of them had significant correlation between beta and mRNA values in the DMC-gene pair analysis. **b** Rank scores of the NAPSA/surfactant metagene per M1-M4 epitype. Values above plot refer to significant p-values derived from the Mann–Whitney test after BH adjustment for multiple testing. *: *p* < 0.05; **: *p* < 0.01; ***: *p* < 0.001. **c** Mean beta values of 60 CpGs around TSSs of seven genes comprised in the NAPSA/surfactant metagene and mean expression of the same seven genes calculated per sample. Values are shown as points per sample and summarized into boxplots per epitype. **d** Scaled mRNA FPKM values of four genes with higher expression in a subset of M4 samples. **e** Selected CpGs close to TSS of genes in the previous panel showing lower DNA methylation and higher expression in a subset of M4 samples. **f** Two examples of candidate genes under epigenetic regulation in LUAD. Samples (columns) are ordered based on Euclidean distance and the complete method. CpGs (rows) are ordered according to genomic coordinates relative to the gene transcription start site (TSS) and the beta values per sample are shown. Bottom panels show mRNA expression of the gene in FPKM for each sample

Of the six expression metagenes analyzed, the NAPSA/surfactant metagene showed the highest proportion of genes within statistically significant DMC-gene pairs from the analysis above (Fig. 3a). Additionally, three of the seven genes included in the NAPSA/surfactant expression signature were among the identified DEGs (Additional file 1: Table S3), and 25% (15/60) of CpGs close to the genes’ TSSs (within a 2.5 kbp genomic window) were DMCs (Additional file 1: Table S4). These results led to the hypothesis of differential epiregulation of this pathway between LUAD epitypes due to their varying rank score sums of mRNA expression (Fig. 3b). Indeed, epitypes varied in gene expression and DNA methylation of NAPSA/surfactant genes both on average (Fig. 3c) and individually per gene (Additional file 2: Fig. S2). It is interesting to note that M3, the cluster exhibiting overall promoter hypermethylation, did not show the highest beta values among LUAD epitypes for CpGs close to NAPSA/surfactant genes. These observations suggest a diversity of regulation of this biological process within the disease, providing partial explanation to the variation in cluster assignment of M2 samples when considering only CpGs in promoters (Fig. 1f).

To identify other genes that might be epigenetically regulated differently across M1-M4 epitypes, we performed a promoter methylation analysis of identified DEGs (Additional file 1: Table S3) with a minimum mean FPKM difference of 30 between epitypes and containing at least one DMC within 6 kbp of the gene’s TSS. Of the 53 genes that fulfilled these requirements, 29 (55%) were differentially expressed in M4 (Additional file 1: Table S3). A closer look at CpGs within the mentioned TSS window revealed epigenetic heterogeneity within M4, with several genes normally epigenetically silenced in LUAD being hypomethylated and expressed in a subset of M4 samples, such as transcription factors (*ASCL1*), calcitonins (*CALCA*), fibrinogens (*FGL1*), and members of the trefoil family (*TFF3*) (Fig. 3d-e).

However, differential DNA methylation of genes within LUAD might not be fully contained within the M1-M4 epitypes detected in this work. To identify other genes possibly under such epigenetic regulation within the disease, we expanded the correlation analysis between gene expression and beta values of close CpGs to all genes in the discovery cohort. After strict filtering of the results, 85 candidate genes remained, including immune response (*C2*, *HHLA2*), cell adhesion (*CEACAM5*), galectin (*LGALS4*), surfactant- (*SFTA2*, *SFTA3*), and transport-associated (*SLC44A4*) genes (Fig. 3f, Additional file 1: Table S5). Of these 85 genes, 70 showed significant variability between epitypes (unadjusted Kruskal–Wallis test, *p* < 0.05) including 51 that were not among the final DEG list.

### Epitypes identified in TCGA LUAD

**Fig. 4:**
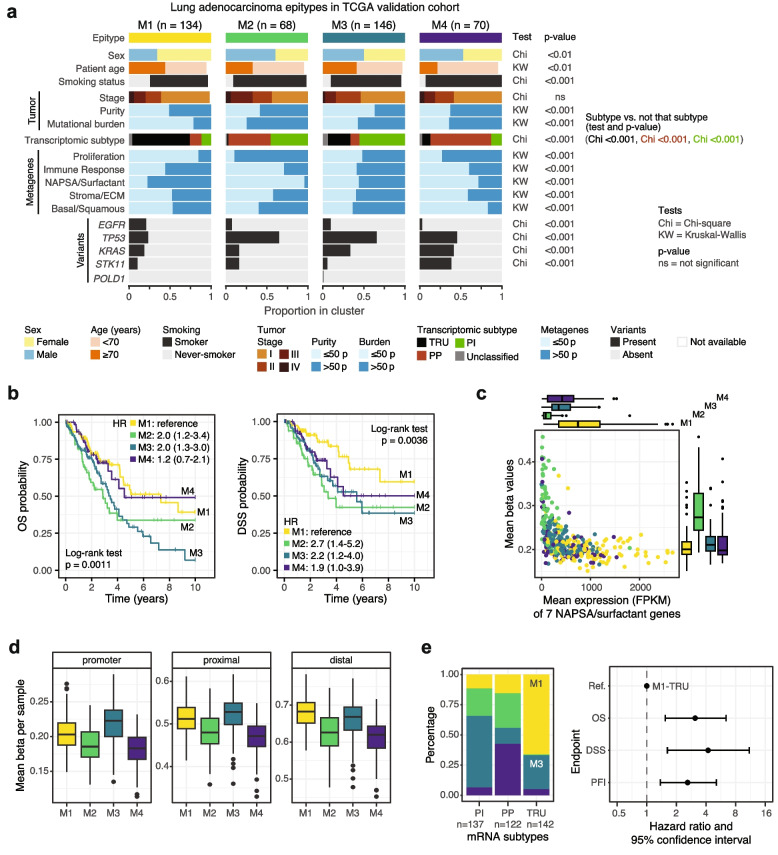
Validation in the TCGA LUAD cohort.** a** Clinicopathological and molecular characteristics summarized per M1-M4 epitype and p-values of tests performed for statistical significance assessment. Numeric variables like tumor purity and metagenes are displayed binarized based at the 50^th^ percentile (50 p) cutoff for each variable. **b** Kaplan–Meier curves using overall survival (OS) and disease-specific survival (DSS) as endpoints. HR = hazard ratio and 95% confidence interval from multivariate Cox regression including patient age at diagnosis and tumor stage as covariates. **c** Mean beta values of 60 CpGs around transcription start sites of seven genes included in the NAPSA/surfactant metagene and mean expression of the same gene set per TCGA sample. **d** Mean beta per sample using all CpGs belonging to specified genomic contexts. **e** Epitype distribution within mRNA subtypes (left) and hazard ratio forest plot for the M3-TRU sample subset compared to the reference (ref.) M1-TRU subset using OS, DSS, and progression-free interval (PFI) as endpoints (right). Ratios were calculated using multivariate Cox regression including patient age and tumor stage as covariates

LUAD samples from TCGA (*n* = 418) were classified into four epitypes with an unsupervised approach following that performed in the discovery cohort (Additional file 2: Fig. S3a). Several characteristics seen in the discovery epitypes were also present in those in the TCGA validation cohort (Fig. 4a). Notably, every key pattern described for M1 was validated in TCGA such as a higher proportion of non-smokers and TRU enrichment. Concordant with general M1 characteristics, exposure to different mutational processes quantified through single base substitution (SBS) patterns known as mutational signatures quantified for TCGA samples revealed higher values of the clock-like signature SBS1 and lower values of signature SBS4, associated with tobacco smoking, in the M1 epitype (Additional file 2: Fig. S3b). Additionally, M1 patients generally showed better expected prognosis than other epitypes (Fig. 4b), in line with its association with the TRU transcriptional subtype and its reported characteristics [6, 7]. Unlike in the discovery cohort but still agreeing with TRU enrichment, M1 in TCGA contained more samples from females than males (Fig. 4a). TCGA samples contain additional information regarding assigned histological subtypes of LUAD, data that were not available for the discovery cohort. Epitypes were not exclusively associated with any histological subtype, but proportions varied across epitypes (Additional file 2: Fig. S3c).

M2 varied the most between cohorts, mainly due to less evident immune patterns in TCGA as exemplified by overall lower values of the immune response metagene in this cohort (Fig. 4a). This can be partly explained by the generally higher tumor purity estimates of TCGA samples (Additional file 2: Fig. S3d) resulting from tumor selection and minimum purity requirements from this initiative, and the difference is especially striking when comparing purity estimates for the M2 epitype (mean 49% in TCGA and 24% in SUH). Cohort differences were also reflected in the transcriptional subtype composition, with much lower proportions of TRU (25.0% in SUH, 1.5% in TCGA) and higher proportions of PP (17.9% in SUH, 51.5% in TCGA) in the M2 epitype. Interestingly, despite the different transcriptional landscape, the identified expression and DNA methylation patterns of genes included in the NAPSA/surfactant signature were also observed in the validation cohort (Fig. 4c). Regarding genomic data, the proportion of *KRAS* variants was lower than expected in this epitype in TCGA (16.2% vs. 46% in SUH). Quantification to exposure to different mutational signatures revealed higher values of signatures SBS2, SBS3, and SBS13 in the M2 epitype (Additional file 2: Fig. S3b). SBS3 is typically associated with homologous recombination deficiency [61] and the other two signatures with activity of cytidine deaminases from the APOBEC family [62].

Unlike M2, M3 repeated most of the patterns observed in the discovery cohort, including the overall promoter hypermethylation (Fig. 4d). Accordingly, this epitype showed higher proportions of the previously proposed [7] CpG island methylator phenotype (CIMP)-high and lower proportions of CIMP-low samples compared to the other epitypes (Additional file 2: Fig. S3e). In this larger validation cohort, M3 patients, together with M2 patients, showed worse prognosis using overall survival and disease-specific survival as endpoints (Fig. 4b). It was interesting to note that, despite being classified as the same transcriptional subtype, M3-TRU samples (*n* = 94) in the TCGA cohort showed significantly worse patient prognoses than M1-TRU samples (*n* = 40) using different available endpoints (Fig. 4e), reflecting biological diversity assessed through epigenomic data within this transcriptional pattern. Further analysis revealed higher scores of the proliferation and basal/squamous metagenes in M3-TRU compared to M1-TRU, as well as lower tumor purity, but no differences in patient age or promoter hypermethylation were observed between the two groups (Additional file 2: Fig. S3f-j).

Lastly, several trends seen in the discovery M4 epitype were observed again in the validation, but some such as the lower immune response or global hypomethylation were less pronounced in TCGA samples (Fig. 4a,d), possibly reflecting tumor inclusion criteria by the initiative. As there are whole exome sequencing data publicly available for TCGA samples, we could investigate proportions of variants in chromatin modifier genes across epitypes and their potential link to the M4 global hypomethylation pattern. Out of 168 genes [63], only three showed statistically significant differences across epitypes (Fisher test, adjusted *p* < 0.05): *CREBBP*, *KDM4D*, and *SMARCA4.* However, proportion of samples with variants were generally low in M4 (< 8%), and such genomic patterns are thus unlikely to be the cause for the pattern observed (Additional file 2: Fig. S3k). Other genomic differences between cohorts included no enrichment for *POLD1* variants (only one TCGA M3 sample had variants in this gene) and more *KRAS* variants than expected in this validation cohort (41.4% vs. 19% in SUH).

### Epitypes identified in the Sandoval validation cohort

**Fig. 5:**
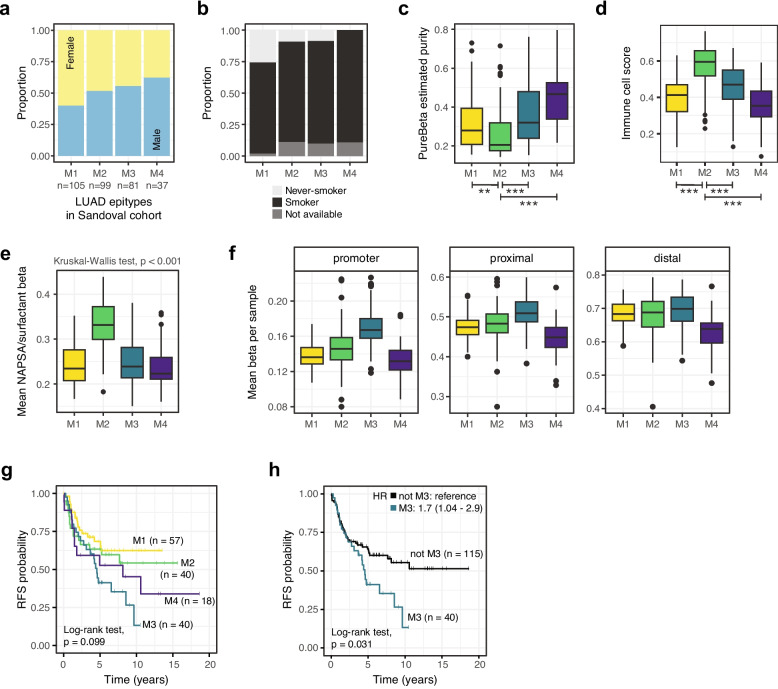
Validation in the Sandoval DNA methylation cohort.** a** Patient sex distribution across epitypes. **b** Smoking history among patients across epitypes. Fisher’s exact test, *p* < 0.001. **c** Tumor purity of samples estimated with PureBeta based on DNA methylation data. Comparisons to M2 values were performed with the T-test and unadjusted p-values are shown under the plot. **: *p* < 0.01; ***: *p* < 0.001. **d** Immune cell scores calculated from imputation of eight immune cell types using MethylCIBERSORT across epitypes. Comparisons to M2 values were performed with the T-test and unadjusted *p* < 0.001 (***) are shown under the plot. **e** Mean beta per sample of 60 CpGs close to the transcription start sites of the seven genes included in the NAPSA/surfactant metagene. **f** Mean beta per sample using all CpGs belonging to specified genomic contexts. **g** Kaplan–Meier curves of M1-M4 epitypes using relapse-free survival (RFS) as endpoint. **h** Kaplan–Meier curves of M3 and not M3 (i.e., M1, M2, and M4 combined) epitypes using RFS as endpoint. HR = hazard ratio and 95% confidence interval from multivariate Cox regression including patient age at diagnosis and tumor stage as covariates. Epitypes failed the proportional hazards assumption (Chi-square test, *p* = 0.01) as evidenced by overlapping curves in the first years after diagnosis

In addition to the TCGA cohort, 322 LUAD samples from the Sandoval cohort were classified into four clusters using the same unsupervised approach applied to tumor purity-adjusted beta values. Despite samples being less stable considering classifications across genomic contexts (Additional file 2: Fig. S4a), several characteristics observed in the SUH discovery and TCGA validation cohorts were also observed in the Sandoval cohort. There was a lack of statistically significant association between M1-M4 epitypes and tumor stage (Chi-square test, *p* = 0.19) or patient sex (Chi-square test, *p* = 0.058), but we observed a tendency for more female patients in the M1 epitype (Fig. 5a). Concerning other patient characteristics, while a higher percentage of non-smokers was confirmed in M1 (Fig. 5b), this epitype did not show higher patient age at diagnosis as seen in the other cohorts (Kruskal–Wallis test, *p* = 0.44).

The M2 epitype had an important immune component in the discovery cohort that was not validated in TCGA samples. In the Sandoval validation cohort, epitypes recapitulated tumor purity patterns seen in the discovery cohort, with lower values in M2 and higher values in M4 (Fig. 5c), indicating immune infiltration differences across epitypes. The immune response expression metagene could not be assessed for Sandoval samples as gene expression data were not available. However, scores for eight immune cell types imputed based on DNA methylation data deconvolution showed a higher combined immune cell score in M2 compared to other epitypes (Fig. 5d), in line with the purity findings. DNA methylation and expression of genes associated with the NAPSA/surfactant metagene were also important for the M2 epitype. While no data were available to confirm expected gene expression patterns, higher DNA methylation of CpGs close to NAPSA/surfactant genes was also observed in the Sandoval M2 epitype (Fig. 5e).

Other epigenetic patterns seen in the discovery cohort and further validated in Sandoval were the general promoter hypermethylation pattern in M3 and the global hypomethylation pattern in the M4 epitype (Fig. 5f), though the latter had not been observed in TCGA. Moreover, most samples with hypomethylation of the *ASCL1* promoter region were assigned to the M4 epitype similar to the other cohorts (Additional file 2: Fig. S4b). Finally, relapse information was available for several patients in the Sandoval cohort. There was weak evidence of differing prognosis among patients assigned to different DNA methylation clusters using RFS as endpoint, with M1 showing better prognosis and M3 worse prognosis (Fig. 5g). In line with was shown for the M3 epitype in previous cohorts, M3 patients showed higher risk of relapse when compared to patients from all other epitypes combined in this second validation cohort (Fig. 5h). Observed survival differences are unlikely to be due to differences in treatment as none of the patients received neoadjuvant therapy and most patients in all epitypes (72–83% of patients with treatment information) also did not receive adjuvant treatment (Additional file 1: Table S1).

### LUAD cell lines mainly resemble the M2 and M3 epitypes

**Fig. 6:**
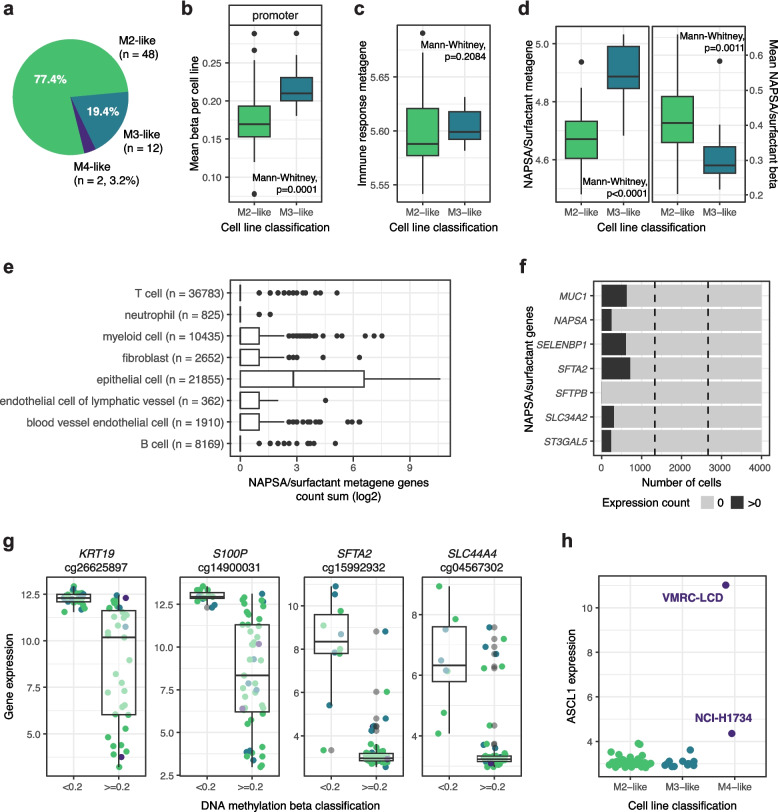
Characteristics of LUAD cell lines classified into the tumor epitypes. **a** Final epitype classification of the 62 cell lines. **b** Mean beta per cell line using all CpGs in gene promoters for cell lines classified as M2- and M3-like. **c** Distribution of the immune response expression metagene score (log10) for cell lines classified as M2- and M3-like. **d** Distribution of the NAPSA/surfactant expression metagene score (log10) for cell lines classified as M2- and M3-like (left) and mean DNA methylation beta values of 60 CpGs around promoters of genes included in this metagene (right). **e** Expression count sum per cell for seven genes in the NAPSA/surfactant metagene from scRNA-seq data of 82,991 cells obtained from lung adenocarcinoma samples. **f** Number of cells expressing any of the seven NAPSA/surfactant metagene genes among 4000 cells originating from equal proportions of three cell lines (NCI-H2228, NCI-H1975, HCC827). Expression was considered as count above 0 (> 0) for a gene. Dashed lines divide the data set in thirds; as expression does not follow dashed lines for any gene, gene expression differs per cell. **g** Examples of genes potentially under epigenetic regulation in LUAD, their expression and DNA methylation beta values of the highest correlated CpG in the 62 cell lines. Colors correspond to DNA methylation classification of cell lines (M2-, M3-, or M4-like). **h ***ASCL1* expression for the 62 cell lines divided by cell line DNA methylation classification

Cell lines established from tumors are commonly used as models for, e.g., drug discovery. To investigate which epitype available LUAD cell lines resembled the most, we correlated DNA methylation beta values of 62 cell lines with median values from the discovery cohort epitypes (Additional file 2: Fig. S5a). Cell lines were classified mainly as M2-like or M3-like, two were considered closer to M4, and no cell line was classified as M1-like (Fig. 6a). Similarly, cell lines clustered mainly with M2 and M3 tumors from the discovery cohort when NMF clustering was performed on all discovery samples together with one cell line at a time (Additional file 2: Fig. S5b). Based on the correlation classification, we found that M3-like cell lines recapitulated patterns identified in the tumor cohorts and exhibited global promoter hypermethylation compared to M2-like lines (Fig. 6b), while there were not enough M4-like samples to assess global hypomethylation.

Gene expression was investigated in the epitypes through metagene scores, and both immune response and NAPSA/surfactant values differed between M2 and M3 in the discovery cohort. In the cell lines, there were no differences regarding immune response, which is consistent with a lack of TME in vitro (Fig. 6c). On the other hand, M3-like cell lines showed higher scores of the NAPSA/surfactant metagene, as well as lower mean beta values of CpGs close to the genes included in it as seen in cohorts used in this study (Fig. 6d). This suggests that epiregulation of the surfactant pathway is intrinsic to LUAD tumor cells and not connected to other cells in the TME. This hypothesis is further supported by that epithelial cells generally express these seven genes to a higher degree than other cells found in lung adenocarcinoma samples, even if the expression itself varies from cell to cell as evidenced by scRNA-seq data of tumors [57] and of cell lines [59] (Fig. 6e-f).

The availability of cell line DNA methylation and gene expression data also allowed us to further investigate the 85 potentially epigenetically regulated genes identified in LUAD tumor cells in the discovery cohort. Genes had to have at least five CpGs with beta values significantly correlated to gene expression values considering the 62 cell lines, and only those with correlation estimates < −0.25 and adjusted *p*-values < 0.01 were counted. Of the 85 candidate genes from the discovery cohort, 33 showed patterns consistent with epigenetic regulation also in analyzed cell lines under these requirements, such as *KRT19* and *SLC44A4* (Fig. 6g, Additional file 1: Table S5). Lastly, the sample with the highest *ASCL1* expression was classified as M4-like, but beta values of CpGs close to this gene did not seem more hypomethylated than other samples with lower gene expression (Fig. 6h, Additional file 2: Fig. S5c).

## Discussion

Molecular subtypes in early-stage, primary LUAD have been proposed based on information from different omics typically obtained by bulk tissue analysis. Importantly, reported molecular mRNA and protein-based subtypes to date represent a mixture of tumor intrinsic and TME features in profiled biopsies. Here we took the novel approach of dividing primary LUAD into four epigenetic subtypes (M1-M4), or epitypes, using DNA methylation data adjusted to focus on methylation patterns of tumor cells. Although we showed that this adjustment reduces the influence of the TME on bulk beta values, we acknowledge that it is still a limited, in silico correction and therefore cannot fully eliminate non-malignant cell contributions, with some residual TME-derived signals likely remaining. Similarly, the adjustment approach does not take into account copy number alterations, a potential source of bias in DNA methylation data. Aiming to extract more biological significance from the available data, we allowed distinct areas of the genome (i.e., the methylation contexts) to participate more evenly in the sample clustering—another distinction from previous DNA methylation studies in lung cancer (e.g., [8, 52]). This is important as CpG behavior in tumors varies according to local CpG density [25] and methylation contexts show distinct properties such as different beta variance profiles. Importantly, sample classification remained constant for most samples in most contexts in all three cohorts used in this work, supporting that different areas of the genome contribute to the identified tumor phenotypes and, critically, that the M1-M4 epitypes can be reproducibly identified. As only one sample per tumor was available in the analyzed cohorts, we could not assess whether multiple epitypes might coexist within individual tumors, nor whether such heterogeneity, if present, would influence tumor biology or patient outcome.

Here we proposed a four-way separation of LUAD based on DNA methylation, contrary to the three CIMP clusters seen in other important works [7, 11, 12], and identified a clear association only between CIMP-High and the M3 epitype. We had previously suggested an optimal number of four LUAD DNA methylation subgroups (ES1, ES2, ES4, ES5) [8] based on unadjusted data from a cohort partially overlapping with the SUH discovery cohort used here. In our previous publication, ES5 showed the same characteristics as M1 (e.g., an association with patients without a smoking history), but this subgroup was also associated with higher immune/stromal infiltration, which in this work appeared as an M2 trait. Interestingly, ES5 samples were split mainly between M1 (*n* = 12) and M2 (*n* = 8), and ES5-M2 samples were more proliferative and immune active compared to ES5-M1 samples based on metagene analysis. ES4, marked by promoter hypermethylation, had samples mainly in M3 (*n* = 10, the only ES subgroup in this epitype) and in M2 (*n* = 9). Like in this work, ES4-M3 samples showed both more promoter hypermethylation and higher NAPSA/surfactant metagene scores than ES4-M2 samples. Taken together, dividing samples with tumor purity-adjusted DNA methylation data appears to sharpen biological differences between clusters, illustrating how omics readouts can be obscured by varying malignant/non-malignant cell composition of bulk tumor tissue.

The current study supported the reported existence of two molecular subtypes enriched for the TRU (M1 epitype) and PP (M4 epitype) mRNA-defined tumors as distinct entities also on the epigenetic level, but not for the PI subtype. More specifically, the characteristics of M1 are consistent with reports of a subgroup of LUAD patients that despite a smoking history display a molecular phenotype similar to that observed in non-smoking patients [8, 64, 65], suggesting a less smoking-dependent disease development. Unfortunately, the current study is underpowered and lacks complementary whole genome sequencing data to analyze whether the proposed genetic subtypes of lung cancer in never-smokers [66] represent specific epigenetic subsets of M1. Conversely, focused comparisons between TRU samples allocated to the M1 and M3 epitypes showed significant biological heterogeneity within the TRU mRNA subtype that was also reflected in distinct prognosis between subgroups. The group of patients belonging to the M4 epitype was also readily identified by mRNA, protein, and DNA methylation patterns. Here it is important to remember that, unlike the LUAD epitypes, the mentioned mRNA subtypes were defined based on unadjusted gene expression data, that is, considering both tumor intrinsic and extrinsic components, likely affecting the correspondence between the two classification approaches.

With the advent of immunotherapy, the influence of immune cells in tumors is increasingly studied and treatment-predictive biomarkers are sorely needed. Importantly, the M4 epitype was shown to be immune-cold with higher tumor purity, lower antigen processing and presentation, and lower immune response despite a higher TMB, congruent with what has been reported for the associated proteogenomic subtype 4 [9]. Interestingly, there was also heterogeneity within the M4 epitype as a subset of LUAD samples exhibiting promoter hypomethylation and increased expression of *ASCL1*, a transcription factor commonly more active in neuroendocrine lung cancer, seemed to be present mainly in this epitype. M4 characteristics agreed with what has been previously associated with ASCL1-positive LUAD tumors, such as generally poor immune cell infiltration [67]. Presence of heterogeneity within our proposed epitypes suggests that additional epigenetic substructure may be present in LUAD. However, resolving more refined epitypes is challenging because even sizeable cohorts become underpowered after sequential subsetting. Large, well-annotated multiomics LUAD data sets remain scarce, and properly evaluating potential sub-epitypes will have to await the availability of such comprehensive data sets. This limitation also extends to evaluating sex-specific epigenetic differences across the proposed epitypes, an analysis not performed in the current work that would require accounting for several confounding lifestyle and biological factors that differ between sexes in the context of LUAD [68, 69].

In contrast to M1 and M4, the M2 and M3 methylation subgroups were less aligned to proposed mRNA and protein subtypes, thereby representing a potential new molecular distinction in LUAD. Still, M2 and M3 showed specific characteristics across cohorts with respect to, e.g., expression of a key diagnostic marker of LUAD histology, napsin A (*NAPSA*), included in the NAPSA/surfactant metagene shown to be differentially expressed across epitypes and seemingly a tumor intrinsic trait. Napsin A positivity has been shown to be prognostic in LUAD and linked to longer patient survival and variants in the *EGFR* gene [70]. While this is consistent with findings for M1, it is not the case for M3 patients who had the worst prognosis of all groups, despite that M3 tumors showed higher expression of napsin A compared to M2 and M4 tumors. While lower scores of the NAPSA/surfactant metagene in M2 could be explained by higher promoter DNA methylation of the signature’s genes in this epitype compared to all other epitypes, this is not the case for M4. This suggests distinct regulation of the surfactant metabolism between epitypes not necessarily captured by suggested transcriptomic and proteomic subtypes as these were similar between M2 and M3 in the discovery cohort. Epitypes differed with regards to immune activation with highest scores in M2 and lowest in M4, as briefly discussed above. These patterns were observed both in the SUH discovery cohort through gene expression data and in the Sandoval validation cohort through immune cell deconvolution from DNA methylation, but they were not as pronounced in the TCGA validation cohort influenced by higher tumor cell content in its samples. Here it should be noted that the DNA methylation tumor purity adjustment algorithm applied to all cohorts is designed to remove the influence from non-malignant cells before sample clustering. Still, the resulting M1-M4 epitypes showed distinct immune cell composition, in line with the recently reported diversity of immune evasion mechanisms in LUAD [9, 12]. This heterogeneity may lead to differences in immunotherapy response across epitypes, which likely also vary in their sensitivity to targeted treatments such as EGFR inhibitors, given the varying proportions of tumors affected by relevant genetic variants across epitypes. However, such analyses could not be performed in the present retrospective cohorts, as patients were enrolled before these treatments were implemented for early-stage lung adenocarcinoma.

Lastly, cell lines established from different LUAD tumors were classified into the four tumor epitypes. Here it is important to note that we used a simplified correlation or NMF approach to classify each cell line, and that classifications could potentially differ if other algorithms/data subsets had been used instead. Moreover, classification attempts are inherently challenging, and some degree of methodological bias is unavoidable regardless of the chosen approach. While cell lines are not perfect substitutes for primary tumor samples, they still represent a valuable tool in cancer research and can provide important insight into the disease as they lack the TME compartment found in bulk tissue. This implies that the methylation patterns of cell lines should reflect more the intrinsic tumor cell methylation patterns that our DNA methylation adjustment is aimed towards. Acknowledging the underlying genomic phenotypes of cell lines is important for planning and interpreting experiments conducted in vitro. Most LUAD cell lines included here were classified as M2-like and M3-like based on DNA methylation beta values. This is not to say that cell lines representative of the M1 and M4 epitypes do not exist, merely that they could be less common and were not present among the 62 lung cancer cell lines with publicly available data and included in this work. Indeed, as epitype proportions were quite distinct from those seen in the tumor cohorts, these publicly available cell line data do not reflect the biological breadth of available LUAD tumor data, but specific characteristics of interest can still be identified, e.g., a LUAD cell line expressing *ASCL1* (VMRC-LCD). In addition, many cell lines showed negative correlation to DNA methylation beta values of M1 samples. This finding is consistent with M1 being comprised of less proliferative, less aggressive tumors from which it would be conceptually harder to establish immortalized tumor cell lines. However, the fact that none of the cell lines with *EGFR* variants were classified as M1-like based on DNA methylation values poses the question of whether these cell lines are truly representative of primary M1 tumors when exploring treatment options and resistance mechanisms. Still, M2- and M3-like cell lines seemed to recapitulate patterns observed in tumor samples, namely those associated with epigenetic regulation and expression of genes included in the NAPSA/surfactant metagene, suggesting a tumor intrinsic definition of this trait instead of being defined by other cells in the TME. Lastly, the low representation of M4-like cell lines could also reflect reduced viability of globally hypomethylated tumor cells, a defining M4 tumor trait, or progressive methylation occurring in in vitro culture conditions, possibilities that remain to be addressed experimentally.

## Conclusions

In the current study we have identified four DNA methylation subgroups in LUAD based on a discovery cohort followed by independent validation using tumor purity-adjusted methylation data. Two methylation subgroups closely matched previously reported subtypes derived from global mRNA or protein profiling, supporting these subtypes as molecular entities across biological layers in LUAD. DNA methylation analysis identified two additional subtypes with multiple molecular differences, including likely tumor intrinsic alterations relevant for the surfactant metabolism. Intriguingly, DNA methylation patterns of most available LUAD cell lines appeared closest to these two new methylation subtypes. How this observation translates to or impacts generalizability of in vitro results remains to be determined. Taken together, our work shows that DNA methylation data may be useful to further resolve the heterogeneity within primary LUAD. However, realizing the full potential of DNA methylation profiling in LUAD will require large, well-annotated multiomic cohorts, particularly those with detailed treatment information, to support rigorous hypothesis testing in this malignancy, and such cohorts are currently lacking.

## Supplementary Information

Supplementary Material 1: Table S1: Lung adenocarcinoma samples used in this work. Table S2: Cell lines established from lung adenocarcinoma samples used in this work. Table S3: Differential analyses across DNA methylation epitypes in the SUH cohort. Table S4: Differential analyses of adjusted DNA methylation data across epitypes in the SUH cohort. Table S5: Candidate genes under epigenetic regulation in lung adenocarcinoma identified in the SUH cohort.

Supplementary Material 2: Figure S1: Beta adjustment and sample clustering in the discovery cohort. Figure S2: Seven genes included in the NAPSA/surfactant metagene in the discovery cohort. Figure S3: Clustering of TCGA samples and cluster characteristics. Figure S4: Clustering of Sandoval samples and cluster characteristics. Figure S5: Lung adenocarcinoma cell line classification and cluster characteristics.

## Data Availability

Most data used in the analyses performed in this article are available from original publications. Previously published DNA methylation data for the SUH cohort are available in the Gene Expression Omnibus (GEO) repository, (https://www.ncbi.nlm.nih.gov/geo/query/acc.cgi?acc=GSE60645) [27] and (https://www.ncbi.nlm.nih.gov/geo/query/acc.cgi?acc=GSE149521) [29]. DNA methylation data for the SUH cohort generated for the current study are available in GEO, (https://www.ncbi.nlm.nih.gov/geo/query/acc.cgi?acc=GSE311943) [30]. RNA-seq expression data in FPKM for the SUH cohort generated for the current study are available in ArrayExpress, (https://www.ebi.ac.uk/biostudies/arrayexpress/studies/E-MTAB-16082) [45]. For the TCGA cohort, data can be downloaded through the Genomic Data Commons data portal, (https://portal.gdc.cancer.gov). DNA methylation data for the Sandoval cohort are available in GEO, (https://www.ncbi.nlm.nih.gov/geo/query/acc.cgi?acc=GSE39279) [53]. DNA methylation data for LUAD cell lines are available in GEO, (https://www.ncbi.nlm.nih.gov/geo/query/acc.cgi?acc=GSE68379) [56]. Single cell RNA-seq data part of the Human Tumor Atlas Network are available in the CZ CELLxGENE repository, (https://cellxgene.cziscience.com/collections/efd94500-1fdc-4e28-9e9f-a309d0154e21) [58]. The other single cell RNA-seq data are available in GEO, (https://www.ncbi.nlm.nih.gov/geo/query/acc.cgi?acc=GSE111108) [60].

## Abbreviations

ATAC-seq: Assay for Transposase-Accessible Chromatin using sequencing
CIMP: CpG island methylator phenotype
DEG: Differentially expressed gene
DFI: Disease-free interval
DSS: Disease-specific survival
DMC: Differentially methylated CpG
FPKM: Fragments per kilobase million
GEO: Gene Expression Omnibus
GSOA: Gene set overrepresentation analysis
LC-MS: Liquid chromatography-mass spectrometry
LUAD: Lung adenocarcinoma
NMF: Non-negative matrix factorization
OS: Overall survival
PFI: Progression-free interval
PI: Proximal Inflammatory subtype
PP: Proximal Proliferative subtype
RFS: Relapse-free interval
SBS: Single base substitution
TCGA: The Cancer Genome Atlas
TF: Transcription factor
TFBS: Transcription factor binding site
TMB: Tumor mutational burden
TME: Tumor microenvironment
TRU: Terminal Respiratory Unit subtype
TSS: Transcription start site
