# Supplementary material for: Multiomics assessment of lung adenocarcinoma subtypes defined through tumor purity-adjusted DNA methylation
Source: Genome Med. 2026 Feb 14;18:24. doi: 10.1186/s13073-026-01609-x (PMC12927254; doi:10.1186/s13073-026-01609-x)
Supplement: Supplementary file 2 — Supplementary Material 2: Figure S1: Beta adjustment and sample clustering in the discovery cohort. Figure S2: Seven genes included in the NAPSA/surfactant metagene in the discovery cohort. Figure S3: Clustering of TCGA samples and cluster characteristics. Figure S4: Clustering of Sandoval samples and cluster characteristics. Figure S5: Lung adenocarcinoma cell line classification and cluster characteristics. [file 13073_2026_1609_MOESM2_ESM.pdf]

Multiomics assessment of lung adenocarcinoma subtypes defined through tumor purity-adjusted DNA methylation

Deborah F. Nacer, Elsa Arbajian, Srinivas Veerla, Mattias Aine, Mats Jönsson, Frida Rosengren,
Anna Karlsson, Annette Salomonsson, Sofi Isaksson, Maria Planck, Johan Staaf

SUPPLEMENTARY FIGURES

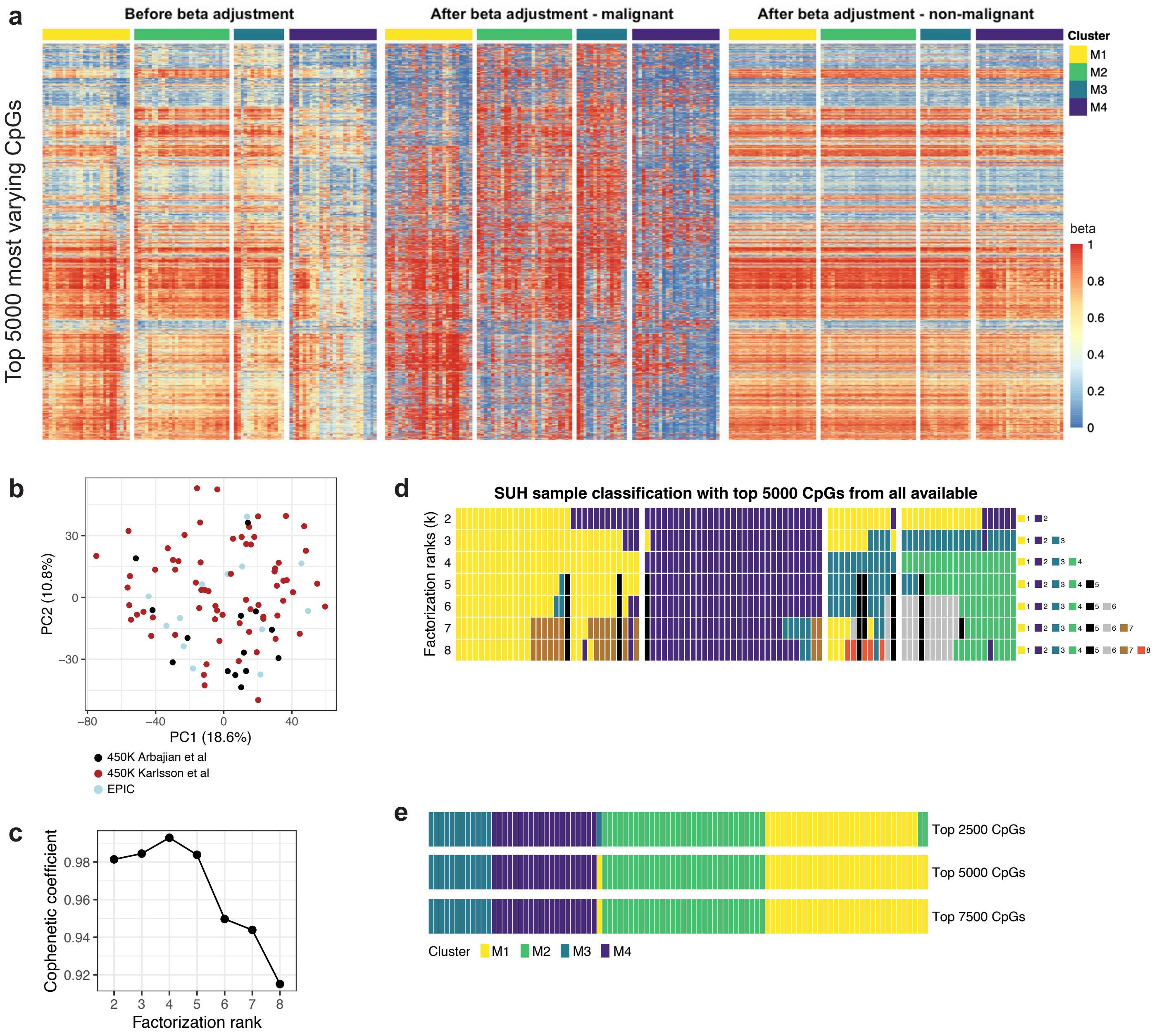

Figure S1. Beta adjustment and sample clustering in the discovery (SUH) cohort. (**a**) Original (left) and adjusted beta values for tumor cells (middle) and non-malignant cells (right) for the 5000 CpGs with highest beta variance (rows) in the 95 samples (columns) of the discovery cohort. Rows and columns are ordered the same in both panels, and they have been ordered using Euclidean distance and the complete method. (**b**) Principal component analysis performed on the 5000 CpGs with highest beta variance in the discovery cohort after beta adjustment and CpG filtering. Performed with the tidymodels R package v1.0.0 after normalizing all predictors (the CpG sites). (**c**) Cophenetic coefficient values for different factorization ranks (target number of clusters) in NMF clustering. (**d**) Sample (columns) classification using different factorization ranks in NMF clustering of the 5000 most varying CpGs in the cohort. (**e**) Sample (columns) cluster assignment using different numbers of most varying CpGs and a factorization rank of 4.

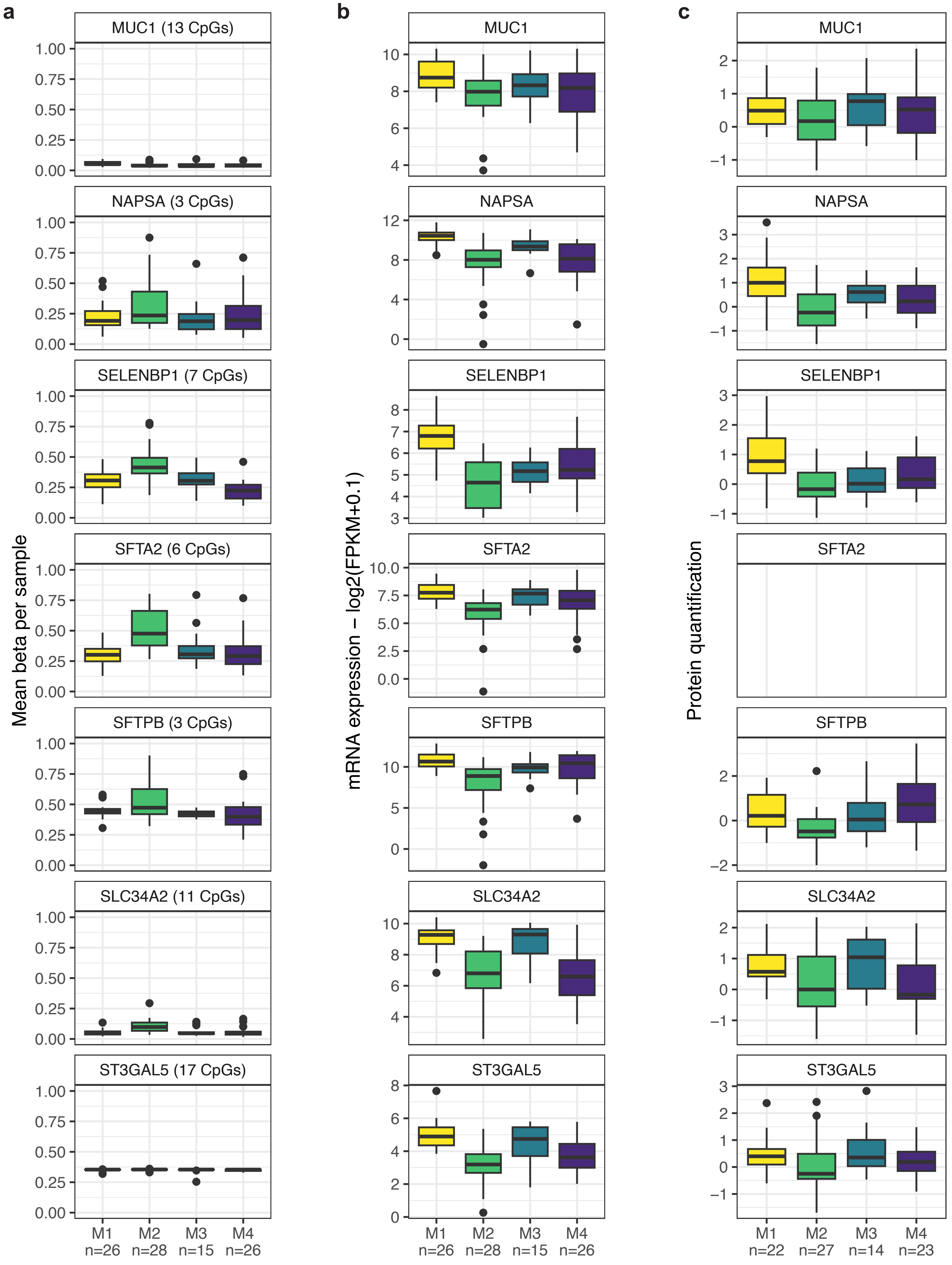

Figure S2. Seven genes included in the NAPSA/surfactant metagene in the discovery cohort. (**a**) Mean DNA methylation beta of CpGs located within a 2.5 kbp genomic window around the gene's transcription start site. For each cluster, *n* samples had data available. (**b**) Gene mRNA expression. (**c**) Protein levels (data for *SFTA2* were not available).

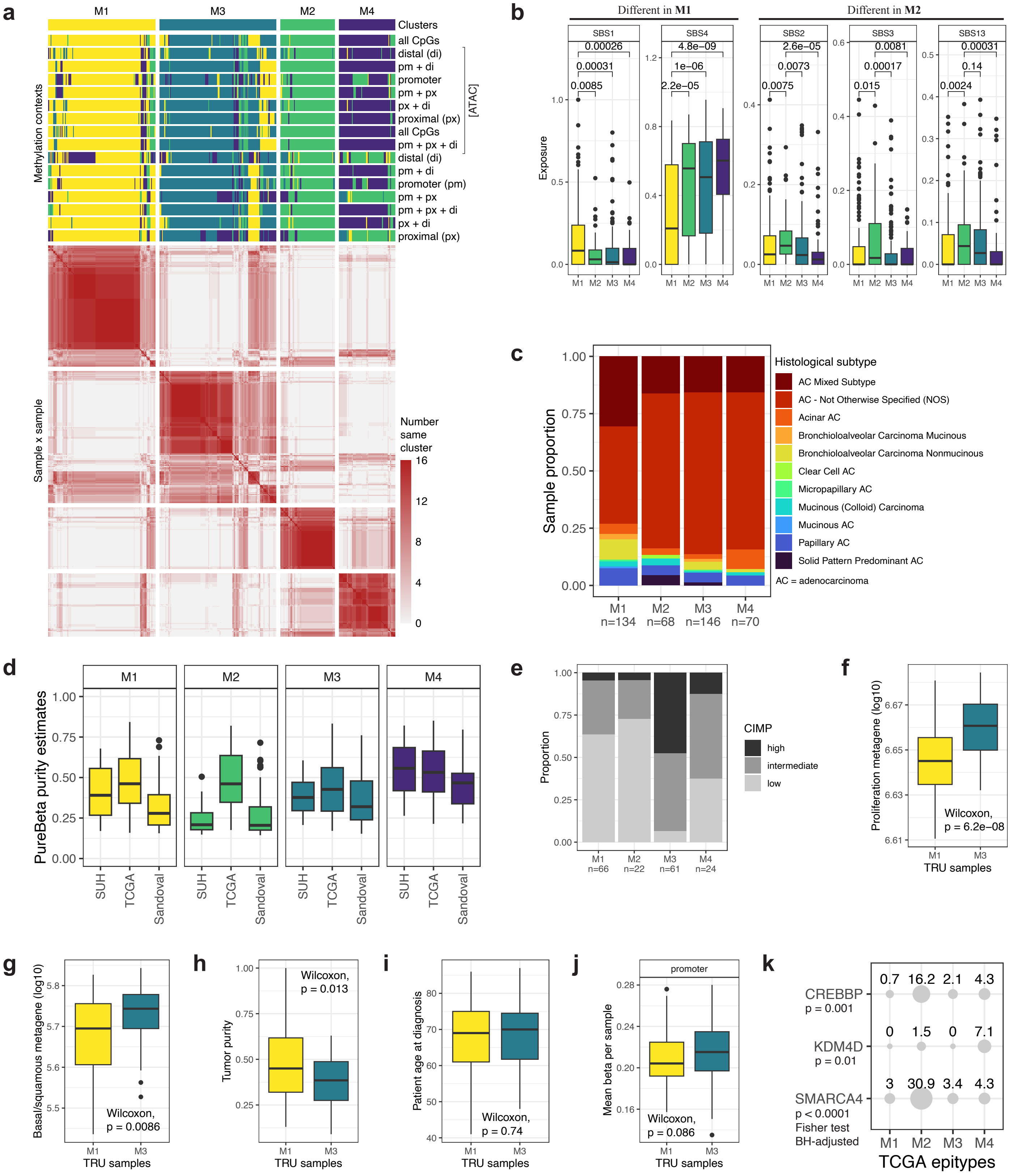

Figure S3. Clustering of TCGA samples and cluster characteristics. (**a**) Sample cluster assignment based on 16 combinations of methylation contexts (above) and how often any two samples were assigned to the same cluster considering all contexts (below). ATAC = CpGs overlapping ATAC-seq peaks, i.e., in regions of open chromatin. (**b**) Distribution of five mutational signatures by cluster. P-values from Wilcoxon test adjusted with the Benjamini-Hochberg method. (**c**) Proportions of assigned histological subtypes of samples by cluster. (**d**) Sample purity estimate calculated with PureBeta [1] divided by cluster and cohort. (**e**) Proportions of proposed CpG island methylator phenotypes (CIMP) [2] by cluster. Only a subset of TCGA samples had such information available. (**f-j**) Comparison between TRU samples classified as M1 (n = 40) or M3 (n = 94) concerning proliferation (f) and basal/squamous (g) expression metagenes, tumor purity (h), patient age at diagnosis (i), and average methylation of CpGs in gene promoters (j). (**k**) Proportion (in %) of samples with variants per gene per epitype.

[1] Sasiain, I., et al. Tumor purity estimated from bulk DNA methylation can be used for adjusting beta values of individual samples to better reflect tumor biology. NAR Genom Bioinform 2024; 6(4):lqae146. **[2]** Cancer Genome Atlas Research N. Comprehensive molecular profiling of lung adenocarcinoma. Nature 2014; 511:543-550.

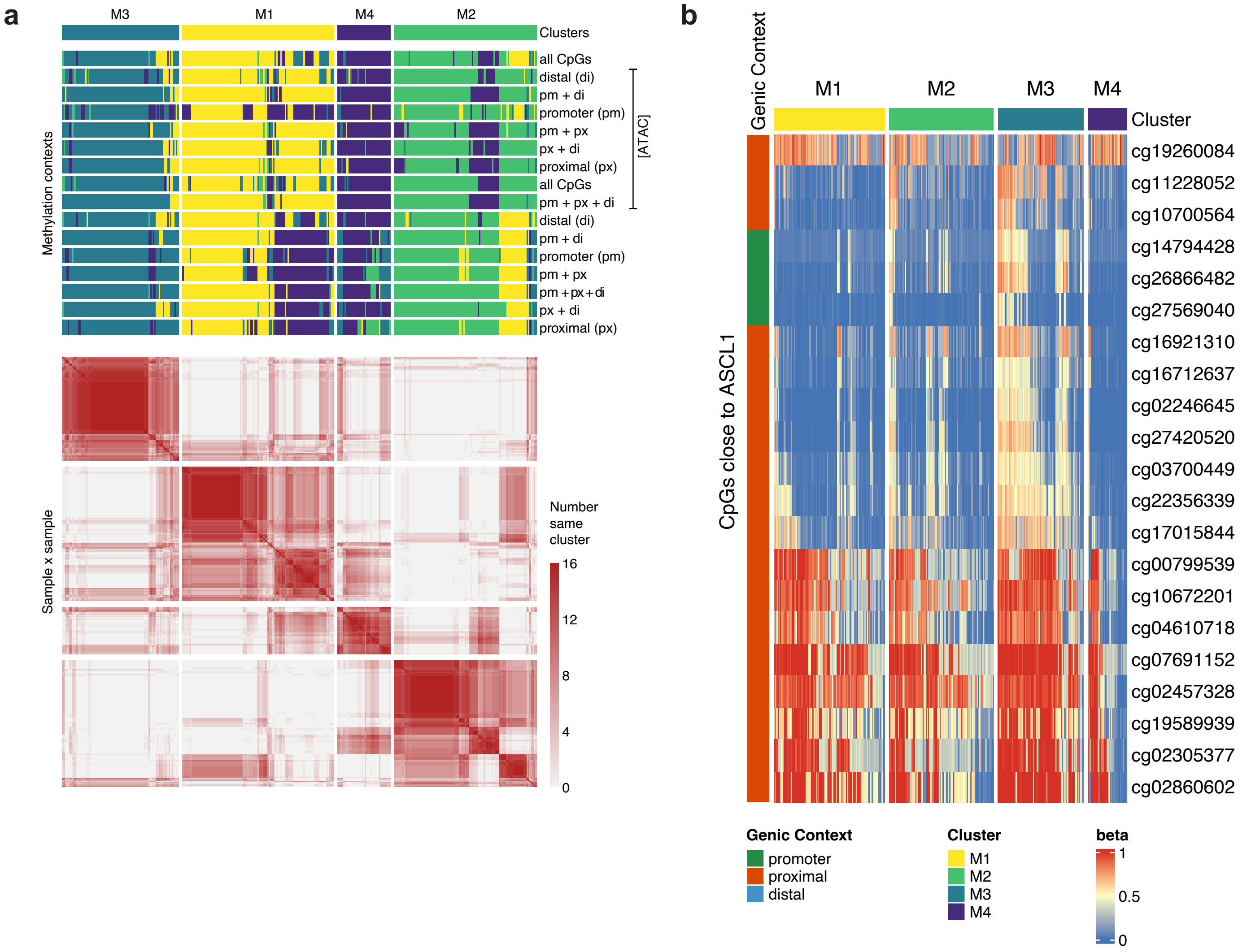

Figure S4. Clustering of Sandoval samples and cluster characteristics. (**a**) Sample cluster assignment based on 16 combinations of methylation contexts (above) and how often any two samples were assigned to the same cluster considering all contexts (below). ATAC = CpGs overlapping ATAC-seq peaks, i.e., in regions of open chromatin. (**b**) Promoter methylation heatmap showing beta values of 25 CpGs close to the *ASCL1* transcription start site (+- 6,000 bp). Samples (columns) are ordered based on Euclidean distance and the complete method within clusters. CpGs (rows) are ordered according to genomic coordinates from lowest to highest.

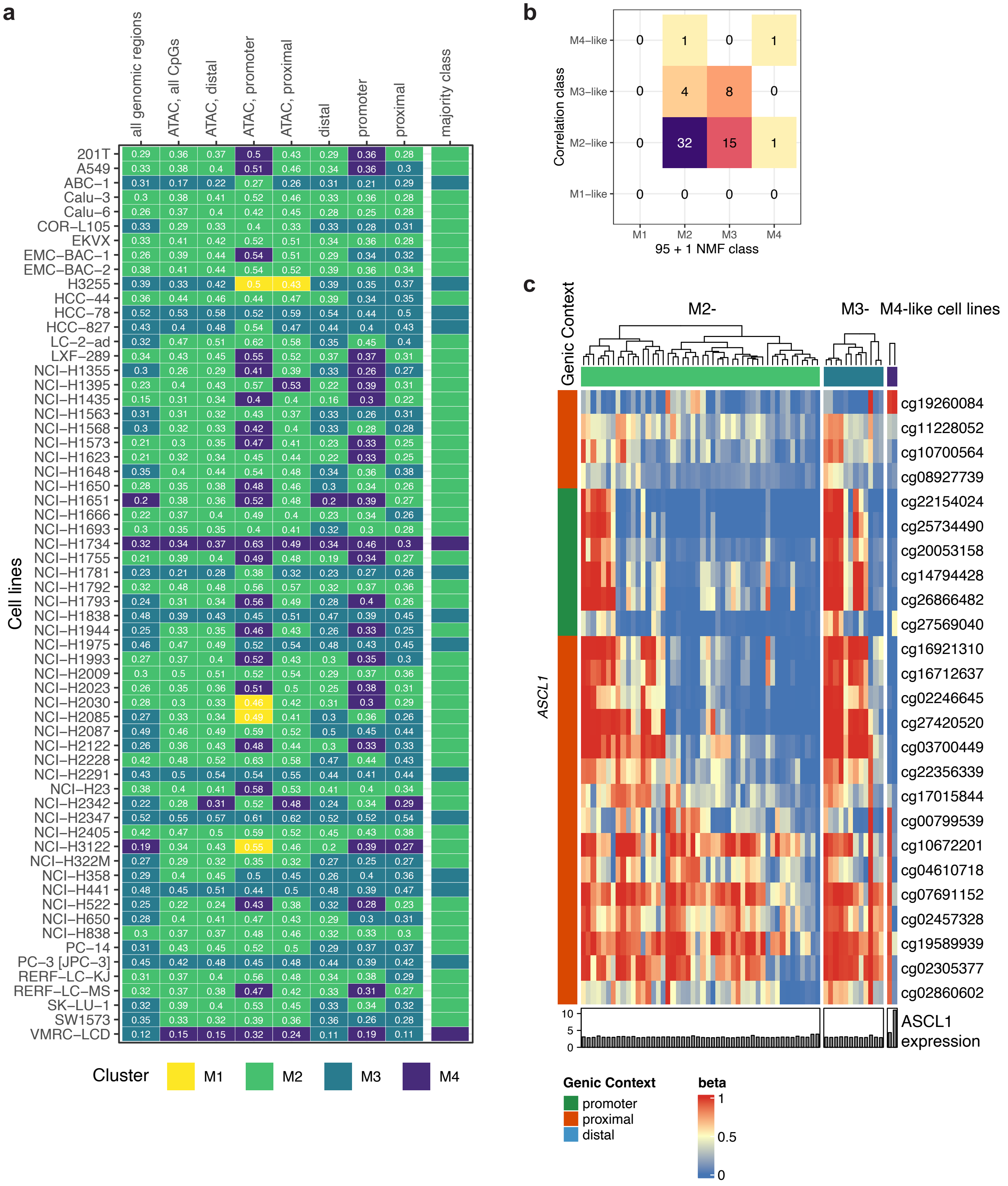

Figure S5. Lung adenocarcinoma cell line classification and cluster characteristics. (**a**) Cluster (colors) with the highest correlation to cell line beta values per CpG context. Spearman correlation (white numbers) was calculated using DNA methylation beta values of the 5000 CpGs per context with the highest beta variance in the discovery cohort. Cell line values were compared to median beta values per cluster. (**b**) Cross-comparison of cell line classification results using the correlation approach from (a) and the approach of performing NMF clustering of all 95 discovery samples and one cell line at a time ("95+1") based on the 5000 most varying CpGs in the discovery cohort and k=4. (**c**) DNA methylation beta values of 25 CpGs (rows) close to the transcription start site (+- 6,000 bp) of the *ASCL1* gene for 62 cell lines (columns) classified into the DNA methylation clusters.
